# The Commoner Diseases of the Eye

**Published:** 1904-06

**Authors:** 


THE COMMONER DISEASES OF THE EYE. HOW TO DETECT AND HOW TO TREAT THEM. By CASEY A. WOOD, C.M., M.D., D.C.L., Professor of Clinical Ophthalmology in the University of Illinois, and Thomas A. Woodruff, M.D., C.M., L.R.C.P., London, Professor of Ophthalmology in the Post-Graduate Medical School, Chicago. Duodecimo, pp. 500, with 250 illustrations, many of them original, of which 7 are colored plates. Chicago: G. P. Englehard & Company. 1904.

In this duodecimo volume of five hundred pages, the authors have undertaken to present ophthalmology to the student and general practitioner in such a way that the more common and important diseases of the eye may be intelligently diagnosticated and treated. The text is clear and concise and is abundantly illustrated by well-executed engravings.

The several chapters include brief references to anatomy and physiology, and a fairly full consideration of refraction and its errors, and of most of the diseases of the eye and its appendages, together with the recognised and most recent methods of examination, diagnosis and treatment. Besides these, there are special and most timely chapters on the commoner pathogenic bacteria that invade the eye, remedies commonly used in ophthalmic practice, the fundamentals of ocular hygiene, headache from eyestrain, ophthalmology in general medicine and surgery, and ocular signs and complications of certain systemic diseases. Throughout the work the needs of the student and general practitioner have been carefully met, and the reviewer believes that, as an introduction to the study of ophthalmology, it has no equal in our language at the present moment. A. A. H.

